# Development and Characteristics of Plant-Based Product Prototypes for Oro-Pharyngeal Dysphagia Diet

**DOI:** 10.3390/foods12030474

**Published:** 2023-01-19

**Authors:** Liene Ozola, Dzaner Shengjuler, Ruta Galoburda, Zanda Kruma, Evita Straumite, Solvita Kampuse

**Affiliations:** Faculty of Food Technology, Latvia University of Life Sciences and Technologies, Riga Street 22, LV-3004 Jelgava, Latvia

**Keywords:** viscosity, consistency, texture, swallowing disorder, proteins, nutritive value

## Abstract

Patients with dysphagia diseases require food with acceptable textural characteristics. Additionally, due to the consumption of smaller portions, these patients receive insufficient amounts of nutrients. Therefore, this study aimed to develop plant-based purée as a meal for an oro-pharyngeal dysphagia (OD) diet, enriched with proteins, fiber and antioxidant vitamins. The suitability of three protein sources—soy protein isolate, whey protein isolate and brown pea protein concentrate—was tested through evaluation of their effect on the rheological properties of protein-enriched plant-based purées for OD diets. Based on the rheological analysis, whey protein was selected for incorporation into the new product formulations. Two prototypes of soups and two prototypes of desserts produced in this study demonstrated acceptable textural properties and high nutritional value.

## 1. Introduction

Many elderly individuals and patients who have experienced cerebrovascular accidents, Parkinson’s and Alzheimer’s disease and neuromuscular disorders suffer from swallowing difficulties (known as dysphagia) [1,2,3]. Oro-pharyngeal dysphagia (OD) is the inability to transport bolus from the mouth to the esophagus. Texture and rheological properties are crucial to facilitate safe movement of food and to reduce the risk of aspiration. It is possible to modify food products by changing their texture, eliminating the need to chew or otherwise orally prepare food. Various strategies can be used in new product development, such as combining different raw materials and thickeners to slow down the passage of food [4]. However, guidelines and standards for development of dysphagia-oriented products vary across the world and even between countries [2,5]. The National Dysphagia Diet (NDD) guide classified the foods according to their viscosity into several groups: thin, nectar-thick, honey-thick and pudding-thick [6]. This guide describes the viscosity of the product, but does not take into consideration other physical properties such as density, flow properties or yield stress [2]. On the other hand, the International Dysphagia Diet Standardization Initiative (IDDSI) described five levels of dysphagia products, with detailed descriptions (from thin to extremely thick) and applicable tests for suitability evaluation, when preparing foods for direct consumption [7]. However, industrial standards for food producers would be greatly appreciated [2].

The studies on texture-modified foods show the suitability of improving the viscosity of various ingredients. Researchers have used hydrocolloids [8,9], gum-based thickeners [4,10], chia seed mucilage [11] and konjac glucomannan [12].

Impairments in swallowing and the resulting psychological problems may reduce food intake, thus leading to malnutrition [1,13]. Therefore, nutritional composition and recognizable sensory properties also play a key role in product development for dysphagia diets. Studies on dysphagia patients indicated an association of OD with a reduced concentration of β-carotene, vitamin C and vitamin E [14]; therefore, the use of fruits and vegetables would supplement the diet with vitamins, minerals and fiber. Additionally, it is well-known that inclusion of fruits and vegetables in a diet helps to prevent chronic diseases [15]. This is closely related to the fact that phytochemicals, including such as polyphenols, carotenoids, and vitamins, have antioxidative power.

On the other hand, protein and fiber are very important in dysphagia diets; therefore, efforts have been made to develop new nutrient-enriched products. Giura et al. [16] used two proteins and four hydrocolloids for the optimization of formulations in the development of protein-enriched vegetable purées, which appeared to be a good source of proteins and antioxidants. Another study developed products, which may be claimed to be “high protein” and “source of fiber,” for dysphagia diets [17]. Proteins used in these formulations may affect the viscosity and texture of the products. Recent research has focused on finding new protein sources with specific properties. Thus, Ang et al. [18] tested novel composite gels containing 13% whey protein isolate and 4% destructured waxy potato starch, varying in NaCl and CaCl and thus affecting ionic strength and gel-forming properties. This may be a prospective approach due to possibilities in the development of various textures, and further, it may be applicable in 3D printing.

Currently, there is limited information on the effect of protein addition into plant-based purées on their textural and rheological properties, which are extremely important in product development for OD patients. On the market, there are only few commercial products for this consumer group. Majority of the special purpose products do not contain fiber, which is important in food transit. There are only few oral nutrition products produced by “Nutricia”, part of Ltd. “Danone” that contain around 3.6 g of fiber per 100 mL of product [19]. The majority of the products are dairy-based and have chocolate, banana or strawberry flavors. This study aimed to develop plant-based purées as meals for oro-pharyngeal dysphagia diets, enriched with proteins, fiber and antioxidant vitamins.

## 2. Materials and Methods

The study was performed in two stages. In the first stage of product development, the impact of protein addition on the textural properties of plant-based purée was evaluated, allowing for the selection of suitable protein sources for OD product development. Then, in the second stage, product prototypes were produced and their quality was evaluated.

### 2.1. Sample Preparation for Rheology Tests

The plant-based purées contained semi-finished ingredients: Jerusalem artichoke (*Helianthus tuberosus*) purée, apple (*Malus domestica* L.) purée, pumpkin (*Cucurbita maxima, Hubbard* group) purée, strawberry (*Fragaria × ananassa*) pulp juice and sea buckthorn (*Hippophae rhamnoides* L.) pulp juice. The raw materials were produced by Ltd. KEEFA, Natural Food Manufacturer (Stelpe, Latvia), from organically grown fruits, berries and vegetables. They were then packaged in bag-in-box bags equipped with valves and stored in a freezer at −18 ± 1 °C. Prior to use, ingredients were defrosted at ambient temperatures for up to 20 h. A purée made of plant ingredients combined with rapeseed oil (3.1 g/100 g), sugar (2.9 g/100 g), L-arginine (1 g/100 g) and L-ascorbic acid (0.1 g/100 g) was used as a control. For other samples, one type of protein per sample was added to enrich their nutritional value, namely:

- whey protein isolate, ESN Beyond Limits | Pro Series (Fitmart GmbH & Co. KG, Elmshorn, Germany)—animal source, protein content 88.5 g/100 g;
- soy protein isolate, ESN Beyond Limits | Raw Series (Fitmart GmbH & Co. KG, Elmshorn, Germany)—vegetal source, protein content 89 g/100 g;
- organic brown pea protein concentrate, 50.0 BP (Aloja-Starkelsen Ltd., Ungurpils, Latvia)—vegetal source, protein content 50 g/100 g.

Due to the lack of legislative regulations regarding the nutrient composition of texture-modified products, the healthcare professionals roughly recommended that protein content be tested in the first stage of the study. Protein additive was kept at 5 and 9 g per 100 g of product. 

The materials were mixed using a blender until a uniform mass was obtained. Samples were prepared in a STEPHAN UMC 5 vacuum boiling kettle (Stephan Machinery GmbH, Hameln, Germany) at 79 ± 2 °C, 0.06 MPa, for 15 min. Individual samples were then poured into 250 mL glass jars, sealed with metal lids and subjected to a secondary heat treatment. This consisted of either pasteurization (P) at 95 ± 2 °C for 15 min in a water bath or sterilization (S) at 115 ± 1 °C for 5 min in an autoclave HST 50/100 (ZIRBUS technology GmbH, Bad Grund, Germany).

The obtained samples varied by protein type (S—soy, W—whey and P—pea; initial letters were used in sample labeling); protein content (5 g or 9 g per 100 g of product, labeled with the respective number) and type of the secondary treatment (P—pasteurization, S—sterilization, labeled with their respective letters in brackets).

### 2.2. Determination of Textural Properties

The texture of the purée was analyzed according to the method described by Gallego et al. [20], with some modifications, using a texture analyzer TA.HD.Plus (Stable Micro Systems Ltd., Godalming, UK) equipped with a 100 kg load cell and a 35 mm back extrusion rig disc probe, A/BE-d35. The pre-test speed was 1.00 mm/s; the test speed was 1.00 mm/s; the post-test speed was 10 mm/s; the penetrating distance was 15 mm and the trigger force was 0.049 N. Purée samples were placed into sample holder cups and leveled to heights of 3 cm. The analyses were completed in triplicate at room temperature (23 ± 1 °C). The maximum positive force represents firmness (N), the positive area below the penetration curve indicates consistency (N s), and the maximum negative force signifies cohesiveness (N) [16].

### 2.3. Determination of Viscosity

The viscosity of plant-based purée for the OD diet was determined by a DV-III Ultra rheometer (Brookfield Engineering Laboratories, Inc., Middleboro, MA, USA) using a T-F spindle attached to a helipath stand to create a helical path through non-flowing material. This was carried out at 20 rpm with five repetitions, at room temperature (23 ± 1 °C).

### 2.4. Determination of Rheological Properties

The shear stress versus shear rate of plant-based purées was analyzed by a MCR 302 rheometer (Anton Paar, GmbH, Graz, Austria) equipped with a Peltier temperature controller, using a cone-plate geometry system (CP50) with a 1° cone angle set at a 0.1 mm gap and a flow ramp of 1–300 1/s at 25 ± 0.1 °C. One and a half teaspoons of purée were placed on the base plate of rheometer and the excess was trimmed after setting a gap. The results obtained by shear stress measurements were fitted to the rheological models (Bingham and Herschel Bulkley) using the RheoCompass 1.25 software (Anton Paar, Graz, Austria). Each sample was analyzed in triplicate.

Bingham model:

σ = σ_0_ + η_∞_*γ, (1)

where σ (Pa) is shear stress, σ_0_ (Pa) is yield stress, η_∞_ (mPa s) infinite shear viscosity and γ (1/s) is shear rate.

Herschel–Bulkley model:

σ = σ_0_ + K*γ^n^, (2)

where σ (Pa) is the shear stress, σ_0_ (Pa) is the yield stress, K(Pa s^n^) is the consistency index, γ (1/s) is shear rate and n is the flow behavior index.

The amplitude sweep test, at a constant frequency (1 Hz) with increasing strain of 0.1–100%, was applied to determine the linear viscoelastic region (LVR). The oscillatory tests were applied to perform a frequency sweep, with strain (oscillating) at 1% and angular frequency at 100–0.1 rad/s. The measurements were completed in triplicate at 25 ± 0.1 °C. The values of storage (G’) and loss (G’’) moduli describe the viscoelastic properties of the purée.

### 2.5. Determination of Target Values for Apparent Viscosity and Textural Properties

Medical experts, working daily with dysphagia patients, were invited to apply their knowledge regarding setting the optimum values for the textural properties of foods intended for OD diet, because the guidelines and standards for dysphagia-oriented products vary across the world, and there are no uniform standards. The determination of target values for viscosity and texture was organized in three independent sessions. Five experts—medical doctors (dietitians and audio speech therapists)—participated in each session. Their task was to select, among the provided samples, the range of suitable textures for IDDSI level 4 OD patients —extremely thick purée [7]. In the first session, commercially available foods of various textures (yoghurt, pudding, three types of purées, ketchup and tomato paste) were screened according to the spoon-tilt and fork-drip tests [7]. Based on the results from the first session, plant-based purée samples of varied textural properties were made in the laboratory. As a result, the marginal samples (minimum/maximum) were identified by the panelists. Then, instrumental measurements of viscosity, firmness, consistency, and cohesiveness were completed for the selected samples, and the target parameters (Table 1), which were further used in the product development were set.

The target values were considered when developing product prototypes.

### 2.6. Preparation of OD Product Prototypes

After completing the first stage of the study, whey protein was selected as the most suitable protein source for new OD product development. All raw materials of plant origin that were used in product development (Table 2) were provided by Ltd. KEEFA, Natural Food Manufacturer (Stelpe, Latvia). The production of prototypes was completed by the same company in industrial settings in 140 kg batches each. Between the first stage of the current study and the production of the product’s prototype, additional work was completed on the product’s formulation development, which was partially published by Ozola and Kampuse [21] and indicated a need to replace some ingredients and use additional additives of vitamin and mineral complexes. As a result, adjustments to the proportions of liquid and thick ingredients were required. Therefore, the decision was made to reduce the protein content by 1 gram in the industrial product prototypes in comparison to the products studied in the first stage. 

The main steps in the production of the product’s prototype were weighing and mixing the ingredients according to the developed formulation (Table 2); vacuum cooking at 78 ± 2 °C, 0.05 MPa for 20 min; adding vitamin and mineral compound premixes (Mg 7.39–9.04 g/kg, Ca 80.78–98.73 g/kg, Fe 0.96–1.18 g/kg, Zn 675.00–825.00 m g/kg, Se 4.86–7.29 mg/kg; vitamins: A 141.84–204.11 mg/kg, D_3_ 2.10–3.02 mg/kg, E 520.41–704.08 mg/kg, C 60.11–73.46 g/kg, B_6_ 404.76–547.62 mg/kg, B_12_ 1.17–1.69 mg/kg, B_9_ 28.90–39.10 mg/kg); stirring at 92 ± 2 °C for 5 min; homogenization; filling into stand-up pouches (200 ± 5 g, each); sterilization at 118 ± 2 °C for 10 min; and, finally, cooling and storing at 20 ± 2 °C until analysis.

### 2.7. Determination of OD Product Prototype Attributes

Three pouches per batch were randomly selected for analysis. The variables and the respective standards and methods are summarized in Table 3.

### 2.8. Data Analysis

Values are expressed as mean ± standard deviation. Data analysis was performed using one-way analysis of variance (ANOVA) followed by Tukey’s test. Differences between samples were considered significant at *p* ≤ 0.05.

## 3. Results and Discussion

### 3.1. Textural Attributes of Plant-Based Purées Intended for OD Diet

**Figure 1:**
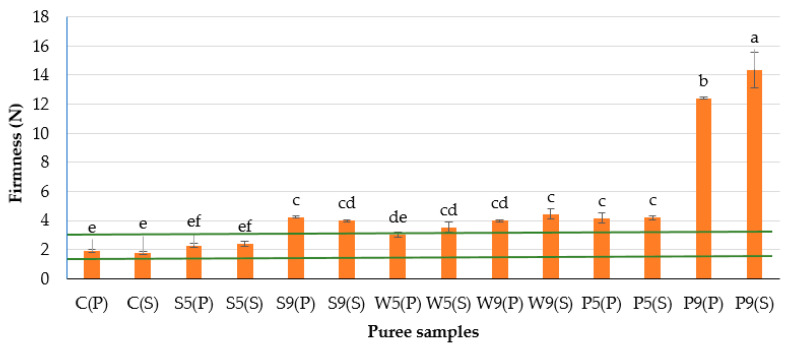
Firmness of the plant-based purées for OD patients. The initial letters in sample abbreviations, C—control (without protein additive), S—soy, W—whey and P—pea protein, represent the samples used in the formulations; the numbers represent the proportions of protein added (5 or 9 g/100 g); the letters in brackets represent the type of secondary treatment (P—pasteurization; S—sterilization). Horizontal lines indicate the target values. Different letters indicate significant differences between samples (*p* ≤ 0.05).

The firmness of the plant-based purée samples (Figure 1) was significantly affected by the type of protein source and the concentration used in the study. The higher the value, the higher the firmness of the product [16]. The type of secondary treatment did not have a significant effect on the firmness of the plant-based purée. Protein was found to harden the texture of the purées. High levels of firmness for the samples with pea protein concentrate made them unsuitable for plant-based purée preparation because they exceeded the target values set by medical experts for patients with swallowing disorders, who need products which can be easily chewed and swallowed. The increased concentration of pea protein from 5 to 9 g/100 g resulted in firmness approximately four-fold higher; the same increase in soy protein additive almost doubled the firmness of the purée, which would cause it to require greater tongue muscle power to initiate swallowing. An increase in whey protein concentration did not have a significant effect on the firmness of plant-based purée.

**Figure 2:**
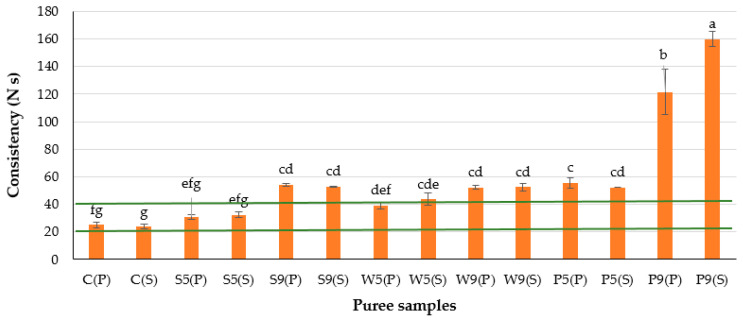
Consistency of the plant-based purées for OD patients. The initial letters in sample abbreviations, C—control (without protein additive), S—soy, W—whey or P—pea protein, represent the samples used in the formulations; the numbers represent the proportions of protein added (5 or 9 g/100 g); the letters in brackets represent the type of secondary treatment (P—pasteurization; S—sterilization). Horizontal lines indicate the target values. Different letters indicate significant differences between samples (*p* ≤ 0.05).

The purées made with soy protein isolate and whey protein isolate at 5 g/100 g were within acceptable limits (20.0–40.0 N s) regarding purée consistency (Figure 2), whereas the same proteins at 9 g/100 g slightly exceeded the set values (Table 1). The higher the consistency value, the thicker the sample [16]. A dramatic increase in consistency was observed for purées made with pea protein concentrate, irrespective of the secondary heat treatment applied. This could be attributed to the higher water binding ability and possible swelling of protein particles and starch, which behave like filling agents and, thus, reduce the food matrix motion, which may result in greater consistency [11,27].

**Figure 3:**
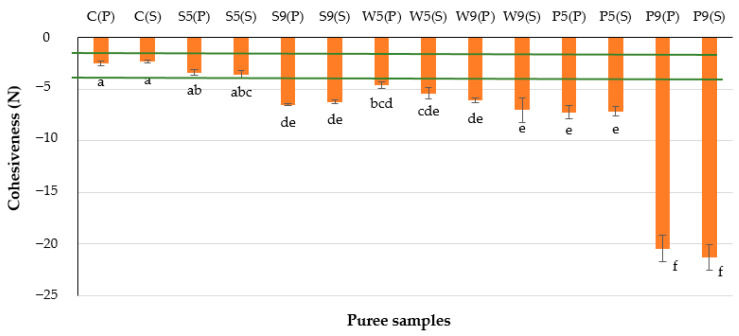
Cohesiveness of the plant-based purées. The initial letters in sample abbreviations, C—control (without protein additive), S—soy, W—whey or P—pea protein, represent the samples used in the formulations; the numbers represent the proportions of protein added (5 or 9 g/100 g); the letters in brackets represent the type of secondary treatment (P—pasteurization; S—sterilization). Horizontal lines indicate the target values. Different letters indicate significant differences between samples (*p* ≤ 0.05).

Increased cohesiveness was observed in purées made with protein additives at 9 g/100 g, irrespective of protein type and heat treatment applied (Figure 3). Purées with soy protein isolate and pea protein concentrate made with 9 g/100 g exhibited two-fold higher cohesiveness compared to the respective purées made with 5 g/100 g protein additive. An increase in whey protein isolate concentration did not cause a drastic rise (*p* > 0.05) in cohesiveness, but for this purpose, the type of heat treatment could be important.

Cohesiveness is related to the product’s ability to keep its structure due to internal stickiness, and it plays an important role in bolus formation [28]. Some products, such as mashed potatoes and heated cereals, exhibit good cohesiveness, whereas others, such as purées, may undergo separation. Many studies have demonstrated the application of various hydrocolloids for the improvement of textural properties [29]. Products with low cohesiveness may cause aspiration due to the formation of more boluses during swallowing [30], whereas highly cohesive products may leave residues in the oro-pharynx, which could also contribute to the risk of aspiration.

### 3.2. Rheological Characteristics of Plant-Based Purées

**Figure 4:**
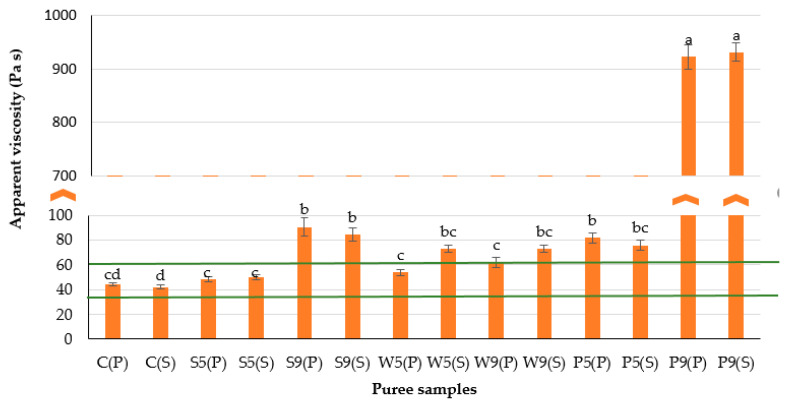
Apparent viscosity of plant-based purée samples enriched with various proteins. The initial letters in sample abbreviations, C—control (without protein additive), S—soy, W—whey or P—pea protein, represent the samples used in the formulations; the numbers represent the proportions of protein added (5 or 9 g/100 g); the letters in brackets represent the type of secondary treatment (P—pasteurization; S—sterilization). Horizontal lines indicate the target values. Different letters indicate significant differences between samples (*p* ≤ 0.05).

The viscosity of purées made with whey protein isolate was stable irrespective of its concentration and the type of heat treatment applied (Figure 4). Plant proteins (soy and pea) at higher concentrations demonstrated a rise in purée viscosity. This may be due to the findings by Sim et al. [31] and Giura et al. [16] that plant proteins, in comparison with animal proteins, have poor solubility, emulsifying, gelling and foaming properties.

**Figure 5:**
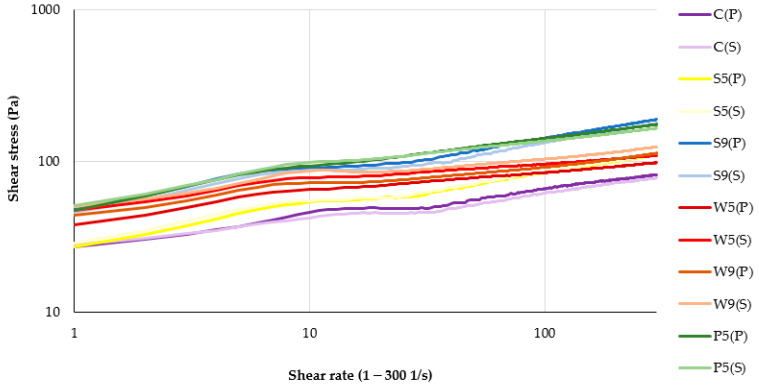
Flow behavior of plant-based purées depending on protein type used and treatment applied. The initial letters in sample abbreviations, C—control (without protein additive), S—soy, W—whey or P—pea protein, represent the samples used in the formulations; the numbers represent the proportions of protein added (5 or 9 g/100 g); the letters in bracket represents the type of secondary treatment (P—pasteurization; S—sterilization). Due to the experimental problems related to the high consistency, it was not possible to measure viscosity for the samples P9(P) and P9(S).

Flow behavior data (Figure 5) allowed for the determination of the yield point, which, according to Nishinari et al. [28], plays an important role in the swallowing process. The yield point for samples containing soy or pea protein was significantly higher at protein additive rates of 9 g/100 g compared to 5 g/100 g. The yield point of plant-based purées enriched with whey protein did not show significant dependency on the protein concentration (Table 4).

The Bingham and Herschel–Bulkley models were used to describe the flow behavior of control and protein-enriched plant-based purée samples. All samples demonstrated shear thinning behavior, which coincides with the findings of Ribes et al. [11], who attributed it to the structural breakdown and increased lining up of molecules caused by applied shear force. The Bingham model better fitted to the experimental data, because in the case of the Herschel–Bulkley model, it was not possible to determine the required parameters for some samples. The use of rheological models allowed us to obtain values of yield stress, which demonstrate the force required to start the flow of the product. The yield stress determined in our study was higher than that reported by earlier studies [32].

The findings are in agreement with the study of Štreimikyte et al. [12], who reported that protein-based beverages suitable for the dysphagia diets of the elderly consisted of yielding, pseudoplastic fluids with σ_0_ > 1 and n < 1. The highest yield stress and viscosity factors were attributed to beverages containing pea protein in comparison to milk protein, possibly due to the excellent water binding capacity of pea protein.

**Figure 6:**
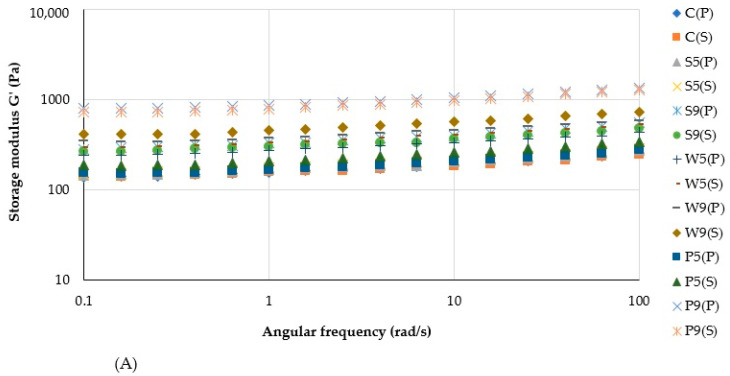

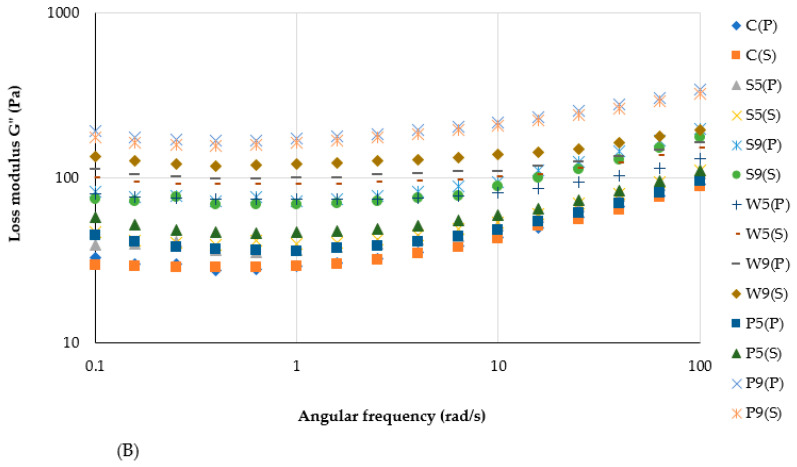
Storage (**A**) and loss (**B**) moduli for plant-based purées. The initial letters in sample abbreviations, C—control (without protein additive), S—soy, W—whey or P—pea protein, represent the samples used in the formulation; the number represents the proportion of protein added (5 or 9 g/100 g); the letters in brackets represent the type of secondary treatment (P—pasteurization; S—sterilization).

Frequency sweep tests (Figure 6) indicated higher values of storage modulus G’ than those of loss modulus G’’ at all conditions, which confirms the gel-like behavior of plant-based purées. Similar to Cuomo et al. [17], who performed rheological assessment of dysphagia-oriented new food preparations, our study revealed the low dependency of the elastic modulus (G’) on frequency, whereas the viscous modulus (G’’) was slightly more frequency-dependent.

The evaluation of textural and rheological properties of protein-enriched plant-based purées led to the decision to select whey protein isolate as a protein additive in the development of product prototypes for OD patients’ diets. Although the results showed that using smaller amounts of soy protein could be just as appropriate as using whey protein isolate to reach textural property goals, it was decided to continue the prototype development using whey protein isolate. 

### 3.3. Characteristics of the Industrially Produced Product Prototypes Intended for OD Diets

Dysphagia patients often eat smaller portions of food, and, thus, receive insufficient nutrients. Therefore, the main target in the development of new product prototypes was adjustment of nutritive value, along with achieving satisfactory rheological attributes. This resulted in two prototypes of soups and two prototypes of desserts being made from local raw materials, intended for OD patients.

The values of viscosity and textural properties (firmness, consistency and cohesiveness) of the industrially produced product prototypes (Table 5) were within the limits set in the first stage of this study by medical experts who work daily with dysphagia patients. Thus, the products would be suitable for patients with swallowing disorders.

The physical and chemical characteristics of industrially produced plant-based purées are summarized in Table 6. The protein content ranged from 9.61 g (D2) to 13.38 g (S2) per 100 g of product. For comparison, the protein content in commercially available adult medical nutrition products is, on average, 12 g per 125 mL (133 g on average) of product, which makes up roughly 9 g per 100 g of products. However, this may vary depending on the intended use. The fat content in the developed product prototypes was, on average, lower than that in commercial products. The total dietary fiber content in the developed products was twice as high as in commercial products. The majority of commercial products do not contain fiber. The content of titratable acids in the produced products ranged from 0.66 g (S2) to 0.88 g (S1) per 100 g of product. The determined chemical composition allowed for the calculation of developed product energy value, which was found to be between 153 kcal (S2) and 162 kcal ((D1) per 100 g. The energy value of commercial products, on average, reaches 300 kcal per 100 g, mainly due to their higher sugar and fat content, making them energy-dense.

The moisture content in the developed products ranged from 71.45 ± 0.05% (D1) to 73.73 ± 0.05% (S2). The samples S1 and S2 had two-fold higher added protein contents, thus requiring more juices instead of purées to be used in the basic formulation (Table 2) to provide suitable rheological properties. The sample S2 had the highest pH (6.22 ± 0.03) and the lowest content of soluble solids (18.2 ± 0.2 Brix%), which, again, can be explained by the formulation of the basic recipe.

The analysis revealed significantly higher total carotene content per 100 g of product in the samples D1 and D2, thanks to higher carrot purée and/or sea buckthorn pulp juice content, compared to other samples. The total phenol content differed among all samples, being the highest in product S2 (199.64 mg TE/100 g). Sample D1 exhibited the highest level of radical scavenging activity of DPPH radical and ABTS radical cations. There were no significant differences (*p* > 0.05) in any of antiradical activities for samples D2 and S1.

Adding the vitamin and mineral compound premix allowed sufficiently high values to be reached, which was not possible using only plant material, as indicated in the previous study [33]. The contents of vitamins B_6_, B_9_, B_12_, C, D_3_ and E and selected minerals are shown in Table 7.

Despite the equal amount of added premix, slight differences were observed among the samples. This could be explained by the differences in the product formulations, as some ingredients are richer in specific vitamins or minerals. Thus, the highest content of vitamin B_12_ was found in sample D2, which also had the highest vitamin B_6_ and D_3_ content. Sample S1 possessed the highest vitamin B_9_ content.

The mineral compound content also varied between samples (Table 7). Overall, the soup samples (S1 and S2) exhibited the highest amount of sodium due to the added salt. The sample S2 differed from other samples, with a higher content of Na, Zn, Ca and Mg.

## 4. Conclusions

After evaluation of the impact of protein additives on plant-based purées, it can be summarized that the purées made with soy protein isolate and whey protein isolate at 5 g/100 g were within acceptable limits regarding their apparent viscosity, consistency, and cohesiveness. The viscosity of purées made with whey protein isolate was stable irrespective of its concentration and the type of the heat treatment applied in the current study. The adopted rheological method allowed us to identify the most suitable protein to be added to plant-based systems, yielding potential prototypes with expected rheological properties. All of the obtained results led to the decision to select whey protein isolate as a protein additive in the product prototype development of oro-pharyngeal dysphagia patients’ diets.

The industrially produced product prototypes had acceptable textural properties. They had similar protein and fat contents, but two-fold higher dietary fiber content compared to commercially available medical nutrition products. The enrichment with vitamin and mineral complex allowed the necessary daily intakes of all nutrients to be reached with the developed products.

Although the mineral and vitamin contents of these products were within the recommended values, these products could be considered as complimentary nutrition instead of a sole source of nutrition. The lack of legislative regulations and standards stated by the medical field provides very little guidelines for the overall nutritional composition of these types of products; therefore, the differences in nutrient content in the developed product prototypes in comparison to commercially available products were not considered to be a failure of the product prototype development. Moreover, these products could be considered as a new nutritional option; they were designed to be consumed as a meal (soup and dessert), instead of as individual products.

## Figures and Tables

**Table 1:** Minimum and maximum target rheological values of plant-based purées for OD patients’ diets.

Variable	Minimum Value	Maximum Value
Apparent viscosity * (Pa s)	35.0	60.0
Firmness (N)	1.5	3.0
Consistency (N s)	20.0	40.0
Cohesiveness (N)	−1.5	−4.0

* Measured with Brookfield instrument at 20 rpm.

**Table 2:** Formulations of plant-based OD product prototypes.

Ingredients	Ingredient Content per Formulation (%)			
	S1	S2	D1	D2
Apple juice	–	–	10.00	15.00
Apple purée	3.00	–	15.00	7.00
Beetroot juice	19.70	–	–	–
Beetroot purée	20.00	–	–	4.00
Cabbage juice	25.74	46.06	–	–
Carrot purée	3.75	12.00	11.00	18.00
Jerusalem artichoke purée	–	–	26.86	24.86
Potatoes	10.50	15.00	–	–
Raspberry pulp juice	–	–	–	17.00
Sea buckthorn pulp juice	–	–	8.00	–
Strawberry pulp juice	–	–	15.00	–
Other ingredients *	17.31	26.94	14.14	14.14

* Other ingredients for soup samples (S1 and S2)—7 g of whey protein isolate and 1 g of hemp protein, salt and spices; and for dessert samples (D1 and D2), 4 g of whey protein isolate and sugar. All samples were enriched with 6 g of rapeseed oil and vitamin and mineral premix.

**Table 3:** Methods and standards used for plant-based purée prototype quality evaluation.

Variables	Standards or Methods with References
Moisture	ISO 1442:1973
Soluble solids	ISO 2173:2003
Titratable acids	ISO 750:1998
pH	ISO 1842:1991
Total proteins	ISO 20483:2013
Total fat	AOAC 920.39
Total dietary fiber	AOAC 985.29
Vitamin C	Seglina [22]
Vitamins B_6_, B_12_, D_3_, E	LC-FLD liquid chromatography
Vitamin B_9_	Nephelometry
Total carotene	Pohloudek-Fabini and Beirich [23]
Mineral compounds Zn, Fe, Cr, Ca, K, Mg, Na, Cu	Inductively coupled plasma mass spectrometry (ICP-MS)
Total phenols	Singleton et al. [24]
Antiradical activity (DPPH)	Yu et al. [25]
Antiradical activity (ABTS)	Re et al. [26]

**Table 4:** The values of rheological models for protein-enriched plant-based purées.

Samples *	Bingham Model		Herschel Bulkley Model		
	Yield Stress, σ_0_ (Pa)	Infinite Shear Viscosity, η_∞_ (Pa s)	Yield Stress, σ_0_ (Pa)	Consistency Index, K (Pa s^n^)	Flow Behavior Index, n (–)
C(P)	43.78 ± 1.46 f	0.227 ± 0.005 fg	n.d.	n.d.	n.d.
C(S)	40.54 ± 0.67 f	0.215 ± 0.007 fg	n.d.	n.d.	n.d.
S5(P)	50.22 ± 1.69 f	0.363 ± 0.008 e	45.25 ± 1.85 d	1.242 ± 0.012 d	0.754 ± 0.034 cd
S5(S)	51.09 ± 2.06 f	0.366 ± 0.009 e	43.82 ± 1.2 de	1.850 ± 0.011 d	0.680 ± 0.014 d
S9(P)	83.32 ± 1.82 bc	0.632 ± 0.023 a	76.03 ± 2.68 a	1.867 ± 0.021 d	0.783 ± 0.032 c
S9(S)	78.74 ± 3.43 cd	0.562 ± 0.017 b	78.65 ± 3.01 a	0.540 ± 0.007 d	0.997 ± 0.047 b
W5(P)	66.07 ± 2.97 e	0.201 ± 0.005 fg	n.d.	63.545 ± 1.252 a	0.099 ± 0.003 h
W5(S)	77.97 ± 2.03 cde	0.199 ± 0.002 g	40.27 ± 1.39 e	23.23 ± 0.846 c	0.190 ± 0.006 g
W9(P)	70.33 ± 2.06 de	0.225 ± 0.010 fg	55.93 ± 1.81 b	5.404 ± 0.026 b	0.408 ± 0.019 e
W9(S)	81.85 ± 4.07 cd	0.232 ± 0.010 f	78.96 ± 2.1 a	0.711 ± 0.032 d	0.777 ± 0.025 c
P5(P)	94.41 ± 4.51 ab	0.528 ± 0.007 c	17.36 ± 0.69 f	43.662 ± 1.404 b	0.228 ± 0.006 fg
P5(S)	97.83 ± 0.04 a	0.424 ± 0.016 d	51.06 ± 0.37 c	23.254 ± 0.393 c	0.282 ± 0.001 f

* Sample labeling: the initial letter indicated either the control (C) (without protein additive) or protein type (S—soy, W—whey, and P—pea); the protein content (5 g or 9 g per 100 g of product was indicated with the respective number); or the type of secondary treatment (P—pasteurization or S—sterilization was indicated with the respective letter in brackets). The average values of three independent measurements ± standard deviations are reported. Different letters in the columns indicate significant differences between samples (*p* ≤ 0.05). n.d.—not detectable.

**Table 5:** Rheological properties of industrially produced plant-based purées intended for OD patients.

Variables	Soups		Desserts	
	S1	S2	D1	D2
Apparent viscosity * (Pa s)	45.66 ± 4.03 b	55.73 ± 2.67 a	34.86 ± 1.14 c	44.30 ± 1.01 b
Firmness (N)	2.65 ± 0.12 ab	2.95 ± 0.20 a	2.04 ± 0.13 c	2.42 ± 0.09 bc
Consistency (N s)	34.03 ± 0.79 b	38.39 ± 2.46 a	24.77 ± 3.36 d	30.60 ± 1.05 c
Cohesiveness (N)	−3.41 ± 0.17 c	−3.86 ± 0.38 c	−2.33 ± 0.11 a	−2.71 ± 0.08 b

* Apparent viscosity was measured by a Brookfield instrument at 20 rpm. Different letters in the rows indicate significant differences between samples (*p* ≤ 0.05).

**Table 6:** Physical and chemical characteristics of industrially produced plant-based purées intended for OD diets.

Variables	Samples			
	S1	S2	D1	D2
Moisture (%)	73.66 ± 0.05 a	73.73 ± 0.05 a	71.45 ± 0.05 b	71.46 ± 0.08 b
Titratable acids (g/100 g)	0.88 ± 0.0 a	0.66 ± 0.0 c	0.75 ± 0.1 b	0.75 ± 0.0 b
Soluble solids (Brix%)	23.6 ± 1.5 b	18.2 ± 0.2 c	23.9 ± 0.3 b	25.8 ± 0.7 a
pH	4.96 ± 0.01 c	6.22 ± 0.03 a	5.24 ± 0.01 b	5.27 ± 0.01 b
Protein content (g/100 g)	10.82 ± 0.0 b	13.38 ± 1.7 a	9.85 ± 0.6 c	9.61 ± 0.2 c
Fat content (g/100 g)	5.77 ± 0.16 a	5.66 ± 0.04 a	5.65 ± 0.27 a	5.51 ± 0.11 a
Total dietary fiber (g/100 g)	8.96 ± 0.36 a	8.75 ± 0.22 a	8.63 ± 0.25 a	8.47 ± 0.06 a
Energy value (kcal/100 g)	155 ± 5 ab	153 ± 3 b	162 ± 3 a	161 ± 3 ab
Energy value (kJ/100 g)	646 ± 20 ab	638 ± 14 b	677 ± 12 a	673 ± 11 ab
Total carotene (mg/100 g)	0.58 ± 0.01 c	1.34 ± 0.27 b	2.35 ± 0.43 a	2.24 ± 0.02 a
Total phenols (mg/100 g)	151.01 ± 7.31 c	199.64 ± 8.25 a	161.33 ± 5.51 c	183.41 ± 4.83 b
DPPH^·^ (mg TE/100 g)	3.10 ± 0.11 b	2.77 ± 0.01 c	3.27 ± 0.02 a	3.10 ± 0.04 b
ABTS^+^ (mg TE/100 g)	12.78 ± 0.30 c	14.01 ± 0.53 b	21.28 ± 0.22 a	12.91 ± 0.67 c

Different letters in the rows indicate significant differences between samples (*p* ≤ 0.05).

**Table 7:** Contents of the selected vitamins and minerals in industrially produced plant-based purées for OD diets.

Variables	Recommended Content (Min–Max Value) *	Values per 100 kcal of Product Sample			
		S1	S2	D1	D2
		Vitamins			
B_6_ (mg)	0.08–0.50	0.81 ± 0.03 b ↑	0.82 ± 0.02 ab ↑	0.78 ± 0.02 b ↑	0.88 ± 0.03 a ↑
B_9_ (μg)	10.00–50.00	75.63 ± 2.63 a ↑	70.68 ± 0.69 ab ↑	69.8 ± 3.27 b ↑	70.27 ± 0.32 ab ↑
B_12_ (μg)	0.07–0.70	1.97 ± 0.06 c ↑	2.04 ± 0.05 c ↑	2.39 ± 0.12 b ↑	3.54 ± 0.1 a ↑
C (mg)	2.25–22.00	108.4 ± 0.72 a ↑	114.4 ± 2.22 a ↑	97.1 ± 2.83 a ↑	97.2 ± 1.56 a ↑
D_3_ (μg)	0.50–2.50	4.64 ± 0.05 b ↑	4.67 ± 0.23 b ↑	4.98 ± 0.18 ab ↑	5.23 ± 0.02 a ↑
E (mg)	0.50–3.00	2.42 ± 0.04 a	2.21 ± 0.11 b	2.46 ± 0.01 a	2.39 ± 0.08 ab
		Minerals			
Zn (mg)	0.50–1.50	2.14 ± 0.03 a ↑	2.16 ± 0.07 a ↑	1.88 ± 0.05 b ↑	2.13 ± 0.07 a ↑
Fe (mg)	0.50–2.00	3.15 ± 0.09 a ↑	2.91 ± 0.07 b ↑	2.64 ± 0.04 c ↑	3.05 ± 0.09 ab ↑
Cr (μg)	1.25–15.00	3.36 ± 0.15 c	3.93 ± 0.09 b	5.13 ± 0.22 a	3.86 ± 0.13 b
Ca (mg)	35.00–175.00	179.7 ± 0.18 b ↑	195.68 ± 7.48 a ↑	183.45 ± 1.79 ab ↑	178.48 ± 6.27 b ↑
K (mg)	80.00–295.00	181 ± 5.3 a	153.8 ± 5.5 b	131.56 ± 0.8 c	124.38 ± 3.75 c
Mg (mg)	7.50–25.00	33.1 ± 1.29 a ↑	33.84 ± 1.01 a ↑	22.92 ± 0.64 b	24.5 ± 0.53 b
Na (mg)	30.00–175.00	91.79 ± 4.02 b	145.29 ± 4.8 a	6.79 ± 0.02 c ↓	8.71 ± 0.23 c ↓
Cu (mg)	0.06–0.50	0.058 ± 0.002 b ↓	0.045 ± 0.002 c ↓	0.083 ± 0.003 a	0.036 ± 0.001 d ↓

* Data according to the Commission Delegated Regulation (EU) 2016/128. Different letters in the rows indicate significant differences between the samples (*p* ≤ 0.05). ↑ shows that the maximum recommended value has been exceeded. ↓ shows that the minimum recommended value has not been reached.

## Data Availability

Data are presented within article.
